# Targets and Effective Constituents of ZhiziBaipi Decoction for Treating Damp-Heat Jaundice Syndrome Based on Chinmedomics Coupled with UPLC-MS/MS

**DOI:** 10.3389/fphar.2022.857361

**Published:** 2022-04-05

**Authors:** Wen-feng Wei, Hui Sun, Shao-bo Liu, Sheng-wen Lu, Ai-hua Zhang, Wan-ying Wang, Wen-jun Chai, Fang-fang Wu, Guang-li Yan, Yu Guan, Xi-jun Wang

**Affiliations:** ^1^ National TCM Key Laboratory of Serum Pharmacochemistry, Metabolomics Laboratory, Department of Pharmaceutical Analysis, National Chinmedomics Research Center, Heilongjiang University of Chinese Medicine, Harbin, China; ^2^ State Key Laboratory of Quality Research in Chinese Medicine, Macau University of Science and Technology, Taipa, Macau SAR, China; ^3^ National Engineering Laboratory for the Development of Southwestern Endangered Medicinal Materials, Guangxi Botanical Garden of Medicinal Plant, Nanning, China

**Keywords:** chinmedomics, UPLC-MS/MS, ZhiziBaipi decoction, damp-heat jaundice syndrome, metabolic profile, combination mechanism

## Abstract

**Background:** Damp-heat jaundice syndrome (DHJS) is a diagnostic model of traditional Chinese medicine (TCM) that refers to jaundice caused by damp-heat pathogen invasion. DHJS is the most common clinical manifestation of TCM, with yellow skin, yellow eyes and anorexia. ZhiziBaipi Decoction (ZBD) is a classic TCM formula that is effective at treating DHJS and various liver diseases. However, the effective components of ZBD in the context of DHJS and the underlying mechanism are unclear.

**Purpose:** This study of ZBD using the DHJS rat model aimed to elucidate the pathobiology of DHJS and the metabolic targets of therapeutic ZBD, construct the network relationship between the components of ZBD and endogenous biomarkers, and clarify the underlying mechanism of ZBD in preventing and treating DHJS.

**Methods:** Using chinmedomics as the core strategy, an animal model was generated, and the therapeutic effect of ZBD was evaluated based on behavioral, histopathological and biochemical indicators. Metabonomics tools were used to identify biomarkers of DHJS, TCM-based serum pharmacochemistry was used to analyze the effective constituents of ZBD, and chinmedomics technology was used to identify ZBD components highly related to DHJS biomarkers.

**Results:** A total of 42 biomarkers were preliminarily identified, and ZBD significantly affected the levels of 29 of these biomarkers. A total of 59 compounds in ZBD were characterized *in vivo*. According to chinmedomics analysis, the highly correlated components found in blood were isoformononetin, 3-O-feruloylquinic acid, glycyrrhizic acid, oxyberberine, obaculactone and five metabolites.

**Conclusions:** Chinmedomics combined with UPLC-MS/MS was used to study the targets and effective constituents of ZBD for the treatment of DHJS.

## Introduction

Damp-heat jaundice syndrome (DHJS) is recorded in the classic traditional Chinese medicine (TCM) resource Shanghanlun, written by Zhongjing Zhang (150–215 A.D.) (Heng et al., 2020). The clinical features of DHJS are yellow eyes, yellow skin, fever, thirst, yellow urine, constipation, hypochondriac pain, dry mouth and bitterness (Bassari and Koea, 2015; Sun et al., 2018). In the clinic, DHJS often appears in the form of various disease complications, which are difficult to treat clinically (Zhang et al., 2011). TCM attributes the etiology of DHJS to endogenous damp-heat, accumulation in the liver and gallbladder, abnormal bile, and consistent exterior and interior manifestations, and these characteristics underlie the pathogenesis and syndrome differentiation of DHJS.

ZhiziBaipi Decoction (ZBD), also recorded in Shanghanlun, is one of the most famous Chinese medicine prescriptions. ZBD consists of *Gardenia jasminoides* Ellis, *Phellodendron amurense* Rupr., and *Glycyrrhiza uralensis* Fisch; this prescription has been used to treat jaundice and liver diseases for more than 1000 years. Modern pharmacological research revealed that ZBD has obvious pharmacological effects, such as protecting the liver (Wang et al., 2016a), promoting bile flow (Qian Zhengyue and Cheng, 2016), inhibiting fibrosis (Chen et al., 2009) and improving liver function, and has broad application prospects in the treatment of liver disease (Wang et al., 2016b). At present, ZBD is often used clinically to treat DHJS, and its curative effect is well-known (Guangming, 2015; Xiaoyan, 2019). Research on ZBD has mostly focused on verifying the traditional curative effects, and there have been no in-depth studies of its mechanism of action and the relationship between its effective components and DHJS biomarkers.

The ambiguity of TCM **syndromes** and the complexity of TCM prescriptions greatly limit the evaluation of the effectiveness of prescriptions and the identification of the material basis of medicinal effects. Chinmedomics, an emerging discipline in recent years, is an applied science that integrates the theories and techniques of systems biology and serum pharmacochemistry of TCM, resulting in the identification of syndrome biomarkers, the establishment of a prescription effectiveness evaluation system, and the discovery of the material basis of pharmacological efficacy (Wang et al., 2016a). Using metabolomics to discover the biomarkers of symptoms and to elaborate the biological nature of symptoms helps to achieve accurate diagnosis of TCM symptoms. Moreover, the establishment of an efficacy evaluation system based on the biomarkers of symptoms to achieve objective evaluation of the efficacy of prescriptions, and to explain the advantages of prescriptions in regulating the overall function of the body through multiple components and targets to treat polygenic complex diseases, and to avoid the biased evaluation of the efficacy of traditional Western medicine prescriptions by pathological methods (Han et al., 2020, Zhang et al., 2015).

In this study, the ZBD was used as the research object, and UPLC/MS technique was used to analyze all the metabolites and then illustrate the pathological mechanism, serum metabolomics method was used to explore the effect and identify potential therapeutic targets, chinmedomics strategy was used to study the mechanism of ZBD against DHJS.

## Materials and Methods

### Materials and Reagents

Chemicals, including HPLC-grade acetonitrile and methanol, were purchased from Merck (Darmstadt, Germany). Distilled water was obtained from Watson’s Food and Beverage Co., Ltd. (Guangzhou, China). Formic acid and leucine enkephalin were obtained from Sigma-Aldrich (St. Louis, MO, United States). Three crude drugs, Gardeniae Fructus [Rubiaceae, *Gardenia jasminoides* Ellis] (Zhi-Zi, ZZ), Phellodendri Amurensis Cortex [Rutaceae, *Phellodendron amurense* Rupr.] (Guan-Huang-Bai, GHB), and Glycyrrhizae radix et rhizoma [Leguminosae, *Glycyrrhiza uralensis* Fisch.] (Gan-Cao, GC), were purchased from Harbin Tongrentang Drug Store (Harbin, China) and authenticated by Xijun Wang, Department of Pharmacognosy of Heilongjiang University of Chinese Medicine.

### Experimental Animals

Male SPF-grade Wistar rats weighing 220 ± 20 g were purchased from the Experimental Animal Center of Heilongjiang University of Traditional Chinese Medicine (Harbin, China) under certificate number SCXK (Liao) 2020-1003. The rats were housed in an animal room at 24 ± 2°C with 50 ± 5% relative humidity and a 12 h/12 h light/dark cycle with free access to food.

### Experimental Instruments

The following instruments and software were purchased from Waters (Milford, MA, United States): Waters Acquity^™^ UPLC liquid chromatograph, Synapt^™^ G2-Si-MS/MS mass spectrometer, UPLC BEH C18 column (1.7 µm, 2.1 × 100 mm), MassLynx V4.1workstation, Progenesis QI 2.0 and UNIFI software.

### ZBD Sample Preparation

According to Zhang Zhongjing’s Treatise on Febrile Diseases in the Eastern Han Dynasty, the modern dosage of ZBD is 17.0 g Zhi-Zi, 27.6 g Guan-Huang-Bai, and 13.8 g Gan-Cao decocted in 800 ml water to a final volume of 300 ml. The ZBD decoction was filtered through 100 mesh gauze, and the filtrate was freeze-dried into powder for later use.

### Animal Grouping, DHJS Modeling and ZBD Administration

The rats were acclimated to the environment for 1 week before the experiment. Then, 30 rats were randomly divided into three groups with 10 rats in each group: the control group, model group and ZBD group. Except for the control group, which received saline, the other groups were orally administered Rhizoma Zingiberis extracting solution (0.1 g/kg) and alcohol (12.5%, v/v) at a dose of 0.1 ml/10 g once a day for 14 consecutive days. ANIT olive oil solution was orally administered at 15.6 mg/kg on the 15th day and at 10.4 mg/kg on the 16th day. The ZBD treatment groups were dosed as follows: from the 17th day to the 22nd day, the rats received 9.2 g/kg ZBD daily. On these days, the control and model groups received the same volume of distilled water orally once a day.

### Serum Sample Collection and Preparation

Rats were intraperitoneally injected with 4% pentobarbital sodium solution (1.5 ml/kg), and blood was collected from the abdominal aorta. After incubation at room temperature for 60 min, the blood samples were centrifuged at 4000 rpm for 15 min at 4°C, and the supernatant (serum) was stored in centrifuge tubes at −80°C for later use. To ensure the stability and repeatability of this isolation process, serum samples were selected from each group as quality control (QC) samples, mixed for 1 min, and centrifuged at 13,000 rpm for 10 min to obtain the supernatant.

### Pharmacodynamic Evaluation of ZBD in DHJS Rats

#### Effect of ZBD on Body Weight and Anal Temperature of DHJS Rats

Changes in body weight and anal temperature of rats were tracked and recorded throughout the experimental period, and data were graphed using GraphPad Prism software (version 8.0, CA, United States) to evaluate the effect of ZBD on the body weight and anal temperature of the model rats.

#### Effect of ZBD on Pathological Changes in Liver Tissue in DHJS Rats

Liver tissue samples were fixed in 10% formaldehyde solution for 48 h, embedded in paraffin, sectioned at 5 mm, stained with hematoxylin and eosin (H&E), and observed under an optical microscope to evaluate the effect of ZBD on liver tissue morphology.

#### Effect of ZBD on Biochemical Indexes in DHJS Rat Serum

The rats were fasted for 12 h, and 1 h after the last treatment, blood was collected from the abdominal aorta into a blood collection vessel containing heparin sodium and centrifuged at 3000 r/min for 10 min to collect the supernatant. The levels of malondialdehyde (MDA), adenosine deaminase (ADA), alanine transaminase (ALT), glutamic oxaloacetic transaminase (GOT), alkaline phosphatase (AKP), direct bilirubin (DBIL), total bilirubin (TBIL), total bile acids (TBA), gamma glutamyl transferase (γ-GT), prealbumin (PAB), total superoxide dismutase (T-SOD) and glutathione peroxidase (GSH-Px) in serum from rats in each group were determined according to the kit instructions.

### Serum Sample Preparation for Metabolomics Analysis

In brief, 400 μl methanol was added to 100 μl rat serum, and the resulting mixture was vortexed for 30 s, incubated at room temperature for 30 min, and centrifuged at 13,000 rpm and 4°Cfor 20 min. Then, 400 μl supernatant was removed, vacuum dried at 40°C, and redissolved in 100 μl 50% methanol. The resulting solution was ultrasonicated for 30 min and then centrifuged at 13,000 rpm and 4°C for 20 min. Of the resulting 100 µl supernatant, 4 µl was injected for UPLC-MS/MS analysis.

### UPLC-MS/MS Conditions

Chromatographic separation was performed by a UPLC system (Acquity^™^ UPLC, Waters Corporation, United States) consisting of a binary solvent manager, a sample manager and a column. Chromatographic separation occurred on an HSST3 column (100 mm × 2.1 mm i.d., 1.8 μm). The column temperature was maintained at 40°C, and the flow rate was 0.4 ml/min. The injected sample volume was 2 μl.The optimal mobile phase consisted of a linear gradient elution program of 0.1% formic acid in acetonitrile (solvent A) and 0.1% formic acid in water (solvent B) as follows: 0 min at 1% A; 0–3.0 min at 1–10% A; 3.1–5.0 min at 10–20% A; 5.1–8.5 min at 20–40% A; 8.6–9.5 min at 40–999% A; and 9.6–11.5 min at 99% A.

High-definition mass spectrometry was performed on a SynaptTM G2-Si TOF MS/MS system (Waters Corporation, United States) equipped with an electrospray ionization (ESI) source in both positive and negative ion modes. The optimized conditions were as follows: positive mode parameters: ion source temperature 110°C, capillary voltage 3000 V, cone voltage 30 V, extraction cone voltage 5.0 V, desolvation temperature 350°C, cone gas flow 50 L/h, and desolvation gas flow 800 L/h; negative mode parameters: capillary voltage 2800 V, cone voltage 40 V, and all other parameters the same as in positive ion mode.

### Multivariate Statistical Analysis

MassLynx software was used to collect chromatographic data, and the UPLC-MS/MS data were imported into Progenesis QI software for peak matching, peak extraction, standardization, data reduction and mass spectrum matrix information acquisition. Furthermore, principal component analysis (PCA) and orthogonal partial least squares-discriminant analysis (OPLS-DA) were performed on each group of data by using the EZ info software module. The variable importance in projection (VIP) score of the first principal component in the OPLS-DA model (threshold value >1) was combined with the *p* value of the *t* test (threshold value 0.05) to identify differentially expressed metabolites.

### Identification of Biomarkers and Metabolic Pathways

Metabolite structures were identified using public network databases (HMDB and METLIN), and the biological significance of potential biomarkers was evaluated using the metabolic pathway databases KEGG and MetaboAnalyst, relevant literature, and biochemistry and molecular biology knowledge.

### Effective Constituents Underlying the Therapeutic Effect

#### Sample Preparation for Constituent Analysis

One milliliter of rat serum was added to preactivated SPE column equilibrated with 2 ml methanol and 2 ml water. The column was washed with 2 ml water and 2 ml 30% acetonitrile, and then, the samples were eluted with 2 ml methanol. After the eluent was collected, it was placed in a nitrogen evaporator at 40°C. The resulting dry residue was redissolved in 200 µl 70% acetonitrile, shaken for 30 s, and centrifuged at 13,000 rpm at 4°C for 15 min, and the supernatant was used for LC-MS analysis.

#### Correlation Analysis Between Biomarkers and Serum Constituents

A chinmedomics strategy was efficiently utilized to discover potentially active metabolites that may contribute to the main therapeutic effect of ZBD in DHJS. The blood grouping (spectrum) matrix and biomarker content change (effect) matrix were imported into PCMS software for identification: the spectrum was C1∼C59, and the effect was B1∼B29. The correlation coefficient parameters were set as follows: correlation coefficient 1 was 0.65, and correlation coefficient 2 was 0.85. The heatmap of the correlation analysis was derived after calculation. The basic screening criteria for potential pharmacodynamic substances were an absolute correlation coefficient value |r| greater than 0.65 and an impact value (ratio of the number of biomarkers with an absolute correlation coefficient greater than 0.65 to the total number of biomarkers) greater than 0.15. In this study, we extracted relevant chemical component data when r ≥ 0.85 and identified the individual components using PCMS.

## Results

### Effect of ZBD on the Body Weight and Anal Temperature of DHJS Rats

**FIGURE 1:**
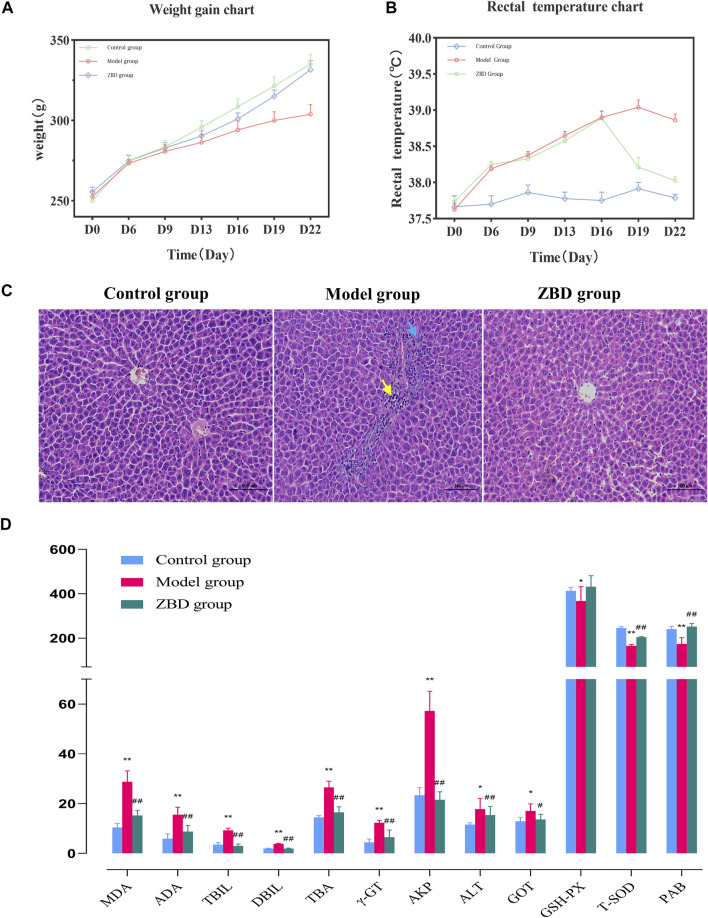
Effect of ZBD on body weight, anal temperature, liver tissue pathology and biochemical indexes in DHJS. **(A)** Average weight in the control, model, and ZBD groups. **(B)** Average rectal temperature in the control, model, and ZBD groups. **(C)** H&E staining of liver tissue for histological evaluation (magnification, 100×). **(D)** Biochemical analysis results for the control, model, and ZBD groups. All the results are presented as the mean ± SD. *n* = 8 for each group. The model group compared with the control group: *p < 0.05, **p < 0.01; the ZBD group compared with the model group: #*p* < 0.05, ##*p* < 0.01.

On day 0 of the experiment, the rats in each group weighed 240 ± 20 g. From day 0–22, body weight in the control group was stable, and growth was normal. Compared with the control group, the model group showed a continuous decrease in weight, which reached the lowest value on the 16th day of the experiment; the difference between groups was statistically significant (*p* < 0.01). From day 16–22, bodyweight remained stable, and the difference between groups remained significant (*p* < 0.01). From day 0–16, the weight of rats in the ZBD group decreased continuously, reaching the lowest value on day 16. From day 16–22, rats given ZBD gained weight, but their body weight remained lower than that of animals in the control group. The results are shown in Figure 1A.

The anal temperature of rats in each group was 37.65 ± 0.09°C on day 0 of the experiment. The anal temperature in the control group was stable from day 0–22. Compared with the control group, the model group showed an upward trend in anal temperature from day 0–22 (*p* < 0.05). The anal temperature in the ZBD group increased (*p* < 0.05) from day 0–16 and decreased (*p* < 0.05) from day 16–22; the results are shown in Figure 1B.

### Effect of ZBD on Pathological Changes in Liver Tissue in DHJS Rats

The H&E staining results showed that liver cells in the control group were normal in shape and orderly arranged with a uniform cytoplasm distribution. There were no pathological changes, such as inflammatory cell infiltration, hepatocyte degeneration and necrosis. In the model group, there was a small amount of localized liver edema at the organ edge. The cytoplasm was enlarged, and the staining intensity was weak. Mild hyperplasia of bile duct cells (blue arrow) and neutrophil infiltration (yellow arrow) were seen in most portal vein areas. Inflammatory cell necrosis was noted in local liver lobules (yellow arrows). Compared with the model group, the ZBD group had significantly reduced liver injury. In the ZBD group, the necrotic area was improved as a whole, the structure of the hepatic lobules was clear, and the hepatic cords were arranged neatly without obvious abnormal lesions. The histopathology and H&E staining results are shown in Figure 1C.

### Effect of ZBD on Serum Biochemical Indexes in DHJS Rats

Previous studies have reported increases in the biochemical markers MDA, ADA, ALT, ALP, DBIL, TBIL, TBA, and γ-GT in clinical jaundice, whereas the levels of PAB, T-SOD and GSH-Px were decreased. In our analysis, compared with the control group, the model group showed significantly increased serum levels of MDA, ADA, ALT, TBIL, DBIL, TBA, γ-GT, ALP and GOT (*p* < 0.01); and significantly decreased levels of PAB, T-SOD and GSH-Px (*p* < 0.05), indicating the successful generation of the model. Compared with the model group, the ZBD-treated group had significantly decreased serum levels of MDA, ADA, ALT, TBIL, DBIL, TBA, γ-GT, ALP and GOT (*p* < 0.01); and no significant difference in ALT. Moreover, PAB and T-SOD levels were significantly increased in the ZBD group compared with the model group (*p* < 0.01), whereas GSH-Px levels showed no significant difference. The experimental results are shown in Figure 1D.

### Multivariate Statistical Analysis of Metabolite Profiles in DHJS Rats

**FIGURE 2:**
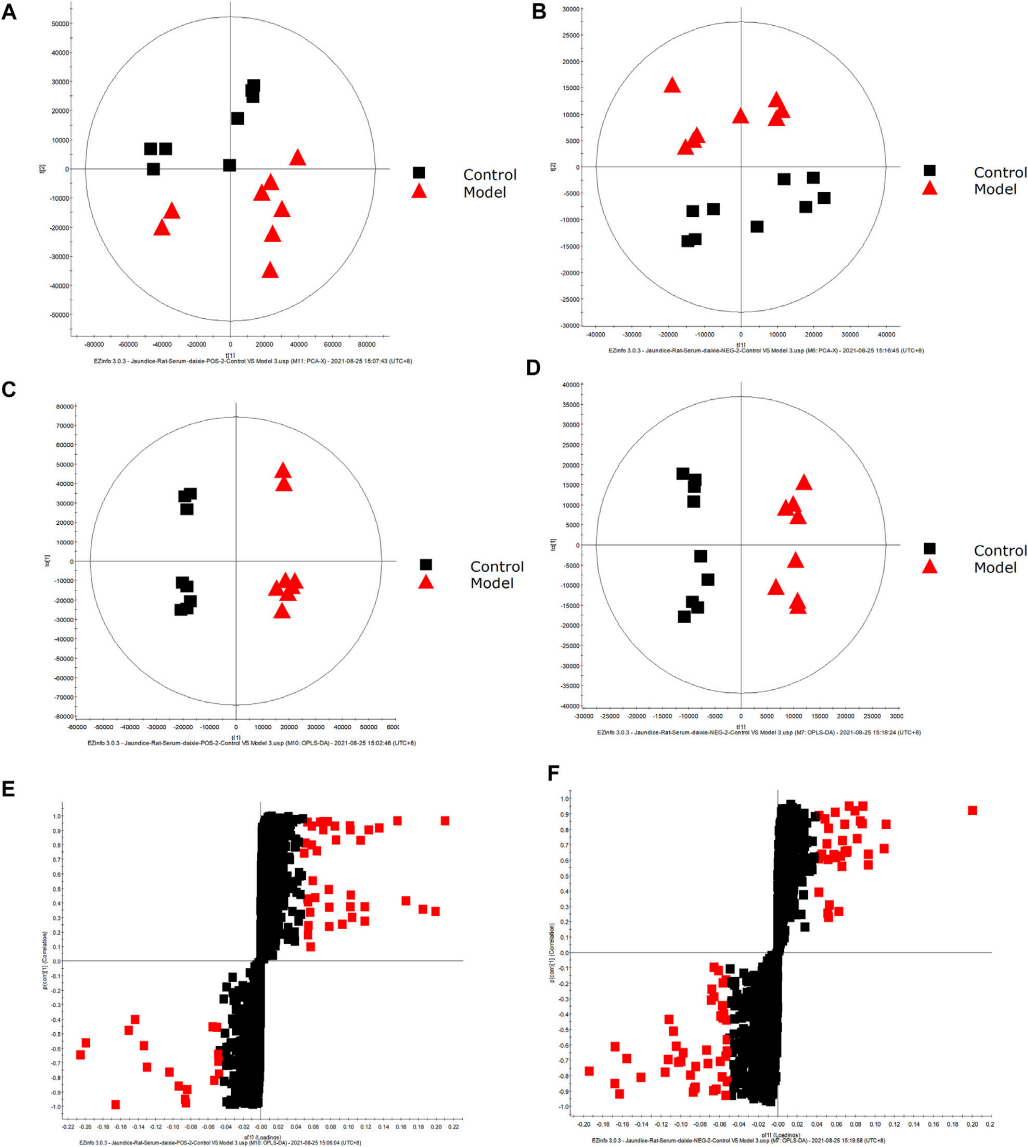
Metabolic profile characterization and Multivariate. **(A)** PCA score plots for the control and model groups in positive mode. **(B)** PCA score plots for the control and model groups in negative mode. **(C)** OPLS-DA score plots for the control and model groups in positive mode. **(D)** OPLS-DA score plots for the control and model groups in negative mode. **(E)** Metabolite biomarkers in the S-plot between the control and model groups in positive mode. **(F)** Metabolite biomarkers in the S-plot between the control and model groups in negative mode.

The obtained UPLC-MS/MS serum metabolic spectrum data were entered into Progenesis QI for chromatographic peak alignment, data normalization, peak extraction and multivariate statistical analysis. PCA of the data was performed by the EZinfo software module, and it was determined whether model creation resulted in changes in endogenous components. PCA was conducted using serum sample data on the 16th day Figures 2A,B. The final model revealed that the data profile of the model group was far away from that of the control group, which indicated a significant change in the metabolic network in the model group. To further distinguish the differences between different groups, supervised OPLS-DA analysis was performed on serum metabolites in the two groups Figures 2C,D.

### Metabolite Identification in Samples From DHJS Rats

A UPLC-MS/MS high-throughput analyzer coupled with Progenesis QI was used to determine the retention time and precise molecular mass and to generate MS/MS data for the structural identification of biomarkers. OPLS-DA was used to analyze the blood metabolic profiles of rats in the model and control groups, and s-plot diagrams, which directly reflect the contribution of each component to the change in the metabolic profile, were created Figures 2E,F to highlight the maximum difference between groups. The selected components were identified by determining their relative molecular weight by primary MS and obtaining their structural fragment information by secondary MS. A total of 42 potential biomarkers were collected using multiple databases, including HMDB, KEGG and METLIN, and the expression levels of each biomarker in the control and model groups are listed in Table 1.

**TABLE 1:** Detailed information on biomarkers tentatively identified by serum metabolomics.

NO.	RT min	M/Z determined	Scan mode	Proposed Composition	Postulated identity	Model vs control	ZBD vs model
1(B1)	0.59	762.537	ESI−	C_44_H_78_NO_7_P	PC(18:4(6Z,9Z,12Z,15Z)/P-18:1(11Z))	↓	↑
2	1.69	219.0774	ESI−	C_11_H_12_N_2_O_3_	5-Hydroxy-L-tryptophan	↓	—
3(B2)	1.71	258.0089	ESI−	C_8_H_7_NO_4_S	Indoxyl sulfate	↓	↑
4(B3)	2.01	613.2982	ESI+	C_33_H_42_N_4_O_6_	D-Urobilinogen	↑	↓
5(B4)	2.06	611.2817	ESI−	C_33_H_40_N_4_O_6_	D-Urobilin	↑	↓
6(B5)	2.19	445.1898	ESI−	C_24_H_30_O_8_	Estrone glucuronide	↓	↑
7(B6)	2.28	199.022	ESI+	C_6_H_8_O_6_	D-Glucurono-6,3-lactone	↑	↓
8(B7)	2.61	512.2682	ESI−	C_26_H_43_NO_7_S	Sulfolithocholylglycine	↑	↓
9(B8)	2.67	423.2726	ESI−	C_24_H_40_O_6_	1b-Hydroxycholic acid	↓	↑
10(B9)	2.83	514.2841	ESI−	C_26_H_45_NO_7_S	Taurocholic acid	↑	↓
11	2.98	448.3064	ESI−	C_26_H_43_NO_5_	Chenodeoxyglycocholic acid	↑	—
12	2.98	464.3015	ESI−	C_26_H_43_NO_6_	Glycocholic acid	↑	—
13(B10)	3.05	371.2558	ESI+	C_24_H_34_O_3_	3-Oxo-4,6-choladienoic acid	↑	↓
14(B11)	3.05	333.204	ESI−	C_19_H_28_O_2_	Etiocholanedione	↓	↑
15(B12)	3.06	148.043	ESI−	C_5_H_11_NO_2_S	L-Methionine	↑	↓
16(B13)	3.08	471.2418	ESI−	C_24_H_40_O_7_S	Chenodeoxycholic acid sulfate	↑	↓
17(B14)	3.1	405.264	ESI−	C_24_H_38_O_5_	7-Ketodeoxycholic acid	↓	↑
18(B15)	3.25	498.2892	ESI−	C_26_H_45_NO_6_S	Tauroursodeoxycholic acid	↑	↓
19	3.25	482.2913	ESI−	C_26_H_45_NO_5_S	Lithocholyltaurine	↑	—
20	3.28	431.2765	ESI+	C_24_H_40_O_5_	Cholic acid	↓	—
21(B16)	3.5	389.2687	ESI−	C_24_H_38_O_4_	12-Ketodeoxycholic acid	↑	↓
22(B17)	3.56	551.3196	ESI−	C_30_H_48_O_9_	Lithocholate 3-O-glucuronide	↓	↑
23(B18)	3.59	583.2558	ESI−	C_33_H_36_N_4_O_6_	Bilirubin	↑	↓
24	3.85	378.2413	ESI−	C_18_H_38_NO_5_P	Sphingosine 1-phosphate	↑	—
25(B19)	3.91	311.2196	ESI−	C_18_H_32_O_4_	13-L-Hydroperoxylinoleic acid	↑	↓
26(B20)	3.99	391.2847	ESI−	C_24_H_40_O_4_	3b,12a-Dihydroxy-5a-cholanoic acid	↑	↓
27(B21)	4.02	562.3144	ESI−	C_26_H_48_NO_7_P	LysoPC(18:3(9Z,12Z,15Z))	↑	↓
28	4.13	285.2065	ESI−	C_16_H_30_O_4_	Hexadecanedioic acid	↓	—
29(B22)	4.29	429.2993	ESI−	C_27_H_42_O_4_	7alpha-Hydroxy-3-oxo-4-cholestenoate	↓	↑
30	4.69	459.2491	ESI+	C_21_H_41_O_7_P	DHAP(18:0)	↓	—
31	4.87	191.0181	ESI+	C_5_H_4_N_4_O_3_	Uric acid	↑	—
32(B23)	4.88	624.3413	ESI−	C_32_H_51_NO_11_	Glycochenodeoxycholic acid 3-glucuronide	↓	↑
33(B24)	4.89	522.3561	ESI+	C_26_H_52_NO_7_P	LysoPC(18:1(9Z))	↓	↑
34(B25)	4.98	471.3468	ESI+	C_28_H_48_O_4_	2-Deoxycastasterone	↓	↑
35(B26)	5.17	599.2885	ESI+	C_34_H_38_N_4_O_6_	Hematoporphyrin IX	↓	↑
36(B27)	5.62	555.2942	ESI+	C_27_H_48_O_8_S	5b-Cyprinol sulfate	↓	↑
37(B28)	5.72	445.3309	ESI−	C_27_H_44_O_2_	7a-Hydroxy-cholestene-3-one	↑	↓
38	6.2	345.2042	ESI−	C_21_H_30_O_4_	Corticosterone	↑	—
39	6.63	756.5532	ESI+	C_40_H_80_NO_8_P	PC(16:0/16:0)	↓	—
40	6.64	184.0741	ESI+	C_10_H_11_NO	Tryptophanol	↓	—
41	6.98	367.158	ESI−	C_19_H_28_O_5_S	Dehydroepiandrosterone sulfate	↑	—
42(B29)	7.79	581.2401	ESI−	C_33_H_34_N_4_O_6_	Biliverdin	↓	↑

↑ and ↓ represent higher and lower level; Model vs control, Model group compared with control group; ZBD vs model, ZBD group compared with model group. No. B1–B29 correspond to Figure 7.

### Metabolic Pathway Analysis in DHJS Rats

**FIGURE 3:**
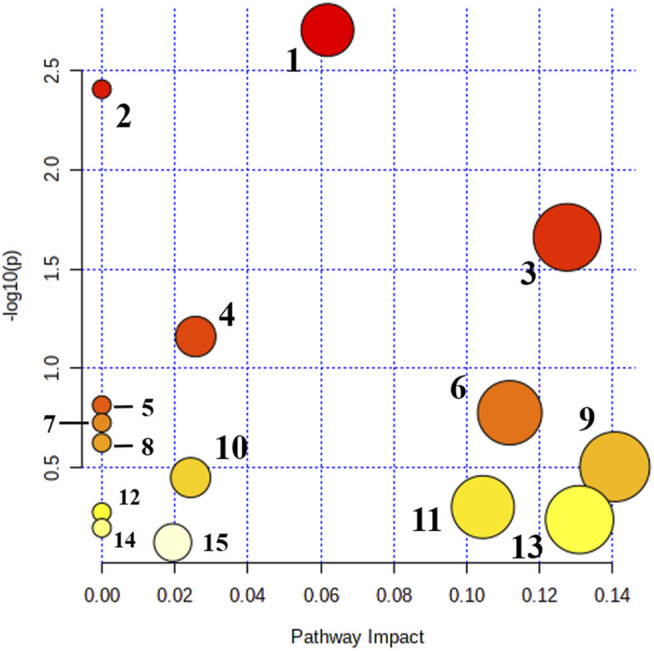
Main metabolic pathways of potential biomakers. 1. Primary bile acid biosynthesis; 2. Linoleic acid metabolism; 3. Porphyrin and chlorophyll metabolism; 4. Steroid hormone biosynthesis; 5. Taurine and hypotaurine metabolism; 6. Glycerophospholipid metabolism; 7. Ascorbate and aldarate metabolism; 8. alpha-Linolenic acid metabolism; 9. Pentose and glucuronate interconversions; 10. Sphingolipid metabolism; 11. Cysteine and methionine metabolism; 12. Arachidonic acid metabolism; 13. Tryptophan metabolism; 14. Aminoacyl-tRNA biosynthesis; and 15. Purine metabolism.

**FIGURE 4:**
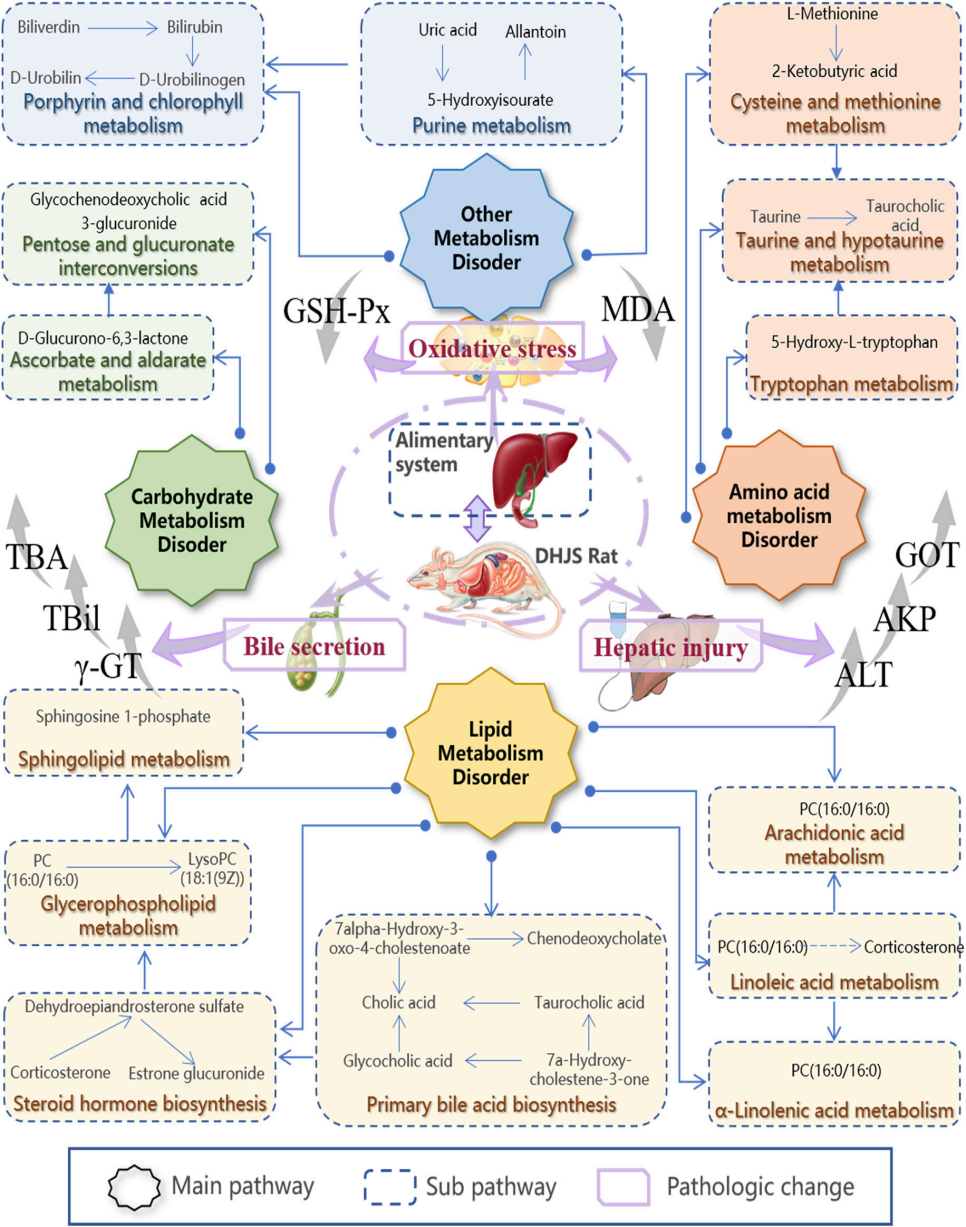
Construction of the altered metabolic network-associated DHJS model based on the KEGG pathway database.

Metabolomics pathway analysis (MetPA) was used to construct and analyze metabolic pathways; the species was set to rat, and the HMDB numbers of the 42 collected metabolites were entered for this analysis. Using topological analysis, the cutoff value of metabolic pathway influence was set to 0.01, and pathways with a value greater than 0.01 were selected as potential key metabolic pathways. A total of 15 metabolic pathways were identified as related to DHJS, including primary bile acid biosynthesis, linoleic acid metabolism, porphyrin and chlorophyll metabolism, steroid hormone biosynthesis, taurine and hypothyroidism metabolism, glycerol phospholipid metabolism, and ascorbic acid and aldose metabolism Figure 3. The networks generated by correlation analysis of metabolite biomarkers and metabolic pathways are described in detail in Figure 4.

### Regulation of Serum Metabolic Disorder by ZBD in DHJS Rats

**FIGURE 5:**
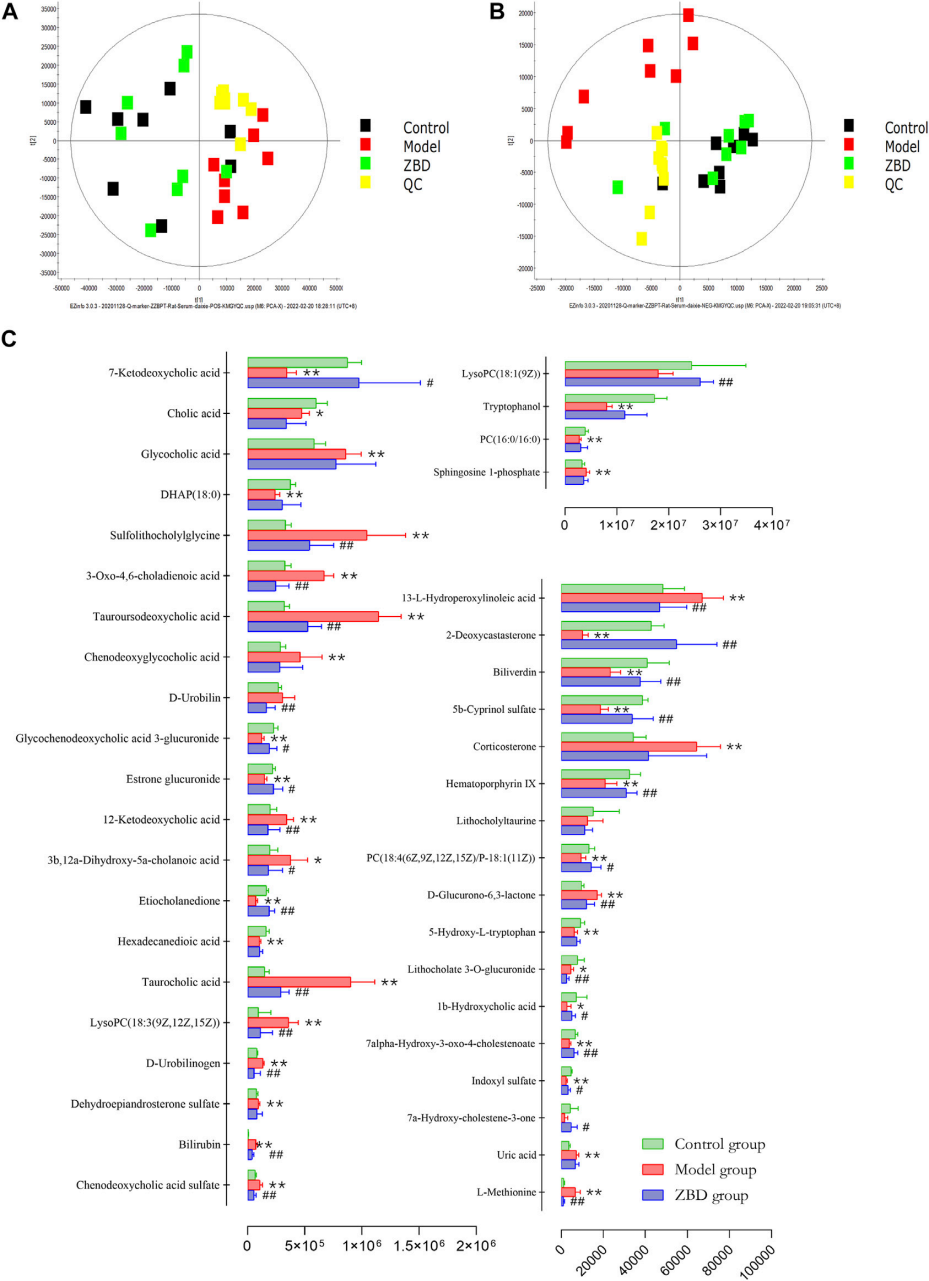
Protective effects of ZBD as determined by metabolic analysis. **(A)** PCA score plots in positive mode. **(B)** PCA score plots in negative mode. **(C)** The relative intensity of serum metabolites associated with ZBD treatment of DHJS. *p <0.05 and **p < 0.01 model group compared with the control group; #*p* < 0.05 and ##*p* < 0.01 ZBD group compared with the model group.

The spatial distribution of PCA scores revealed that rats in the model group could be obviously distinguished from those in the control group, indicating that endogenous regulation alone cannot normalize the metabolic changes in DHJS rats to match metabolite levels in control rats after 21 days. After the oral administration of ZBD, the spatial position of these treated rats was far from that of model rats and close to that of the control rats, which indicates that ZBD was able to normalize the DHJS metabolic profile Figures 5A,B.

### Effect of ZBD on Serum Effector Markers in DHJS Rats

ZBD completely reversed the abnormal levels of 29 biomarkers, including chenodeoxycholic acid sulfate, bilirubin, D-urobilinogen, LysoPC(18:3(9Z,12Z,15Z)), taurocholic acid, etiocholanedione, 3b, 12a-dihydroxy-5a-cholanoic acid, 12-ketodeoxycholic acid, estrone glucuronide, glycochenodeoxycholic acid 3-glucuronide, D-urobilin, tauroursodeoaxycholic acid, 3-oxo-4,6-choladienoic acid, and sulfolithocholylglycine. The relative levels of biomarkers before and after ZBD treatment are shown in Figure 5C.

### Effective Constituents of ZBD in DHJS Rats

**FIGURE 6:**
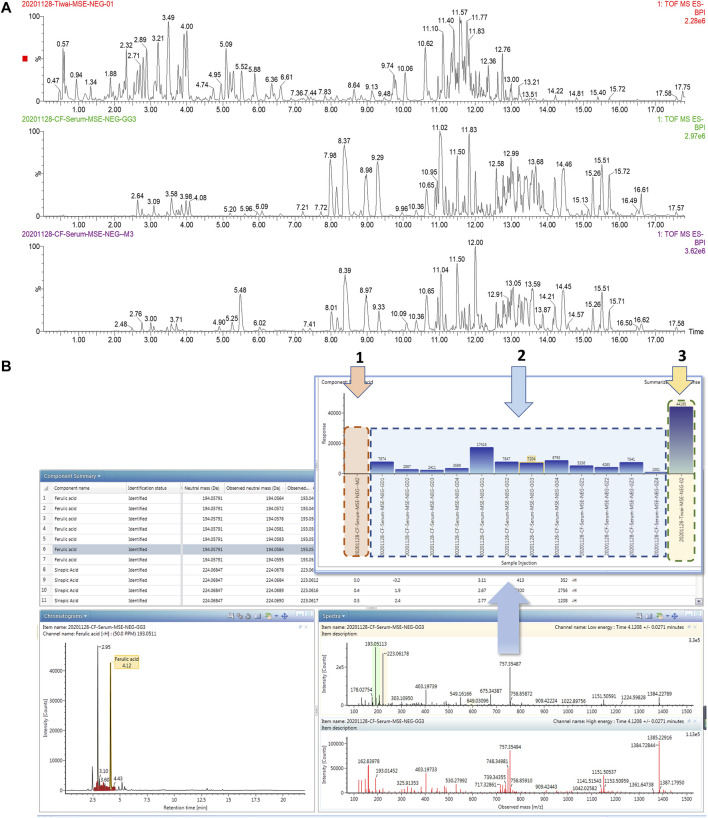
Consultant analysis of ZBD *in vivo* based on UPLC-MS/MS combined with UNIFI ^™^ software. **(A)** Chromatograms of ZBD and serum from the model and ZBD-treated groups. **(B)** The results of matching MS/MS fragment ions and trend plot of the identified absorbed compounds based on the UNIFI database. 1: Relative content of ferulic acid in the (1) model group, (2) ZBD-treated group, and (3) ZBD group.

UPLC-MS/MS and UNIFI™ software were integrated to screen the absorbed constituents and metabolites of ZBD. All data acquired in MS^E^ mode, including those from ZBD-treated *in vitro* samples, model samples and dosed samples, were imported into UNIIF^™^ software. Then, the dataset was processed by the metabolite identification MS^E^ method. Compounds were selected for identification, and the criterion of a 10-fold greater response value in the treated samples than in the control samples was utilized Figure 6. Finally, a total of 59 *in vivo* compounds were tentatively identified as ZBD metabolites by automatically matching the exact mass and MS/MS fragmentation of the compounds in the UNIFI database. Among the 32 prototyp components there are seven components derived from ZZ, nine from GHB and 19 from GC. Syringoside is a common component of ZZ and GHB, and quercetin-3-O-glucopyranoside is a common component of all three herbs. There were 27 drug metabolites, whose precursor compounds were Geniposide, Obaculatone, Isoliquiritigenin, Naringenin, Oxyberberine, Picrocrocinic acid and18β-glycyrrhetinic acid, among which Geniposide, Naringenin, 18β-glycyrrhetinic acid, Naringenin, and Isoliquiritigenin Oxyberberine were metabolized in various ways and could produce phase I and phase II metabolites, while Obaculatone were mainly phase I metabolites. The 32 prototype components and 27 drug metabolites are summarized in Table 2.

**TABLE 2:** Analysis of constituents in DHJS rat serum after the oral administration of ZBD.

NO.	RT/min	Formula	Ion mode	Compound name	Measured mass/Da	Theoretical mass/Da	Error/Da	Fragments	Origin
C1	2.88	C_27_H_32_O_14_	[M−H]−	Naringin	579.172	580.1799	0.82	580/549/417/353/255/191	c
C2	3.08	C_27_H_30_O_15_	[M−H]−	Nicotiflorin	593.1522	594.1618	1.35	593/549/475/137	c
C3	3.31	C_20_H_21_NO_4_	[M+H]+	Tetrahydroberberine	340.1556	339.1494	4.06	340/309/276/147	b
C4	3.49	C_17_H_20_O_9_	[M−H]−	3-O-Feruloylquinic acid	367.1044	368.1115	−2.93	367/191/173/111	b
C5	4.01	C_21_H_22_O_9_	[M−H]−	Isoliquiritin	417.121	418.1308	0.77	417/255/135/119	c
C6	4.11	C_11_H_12_O_5_	[M−H]−	Sinapic acid	223.0622	224.0702	0.69	225/207/189/167/147	c
C7	4.12	C_10_H_10_O_4_	[M−H]−	Ferulic acid	193.0511	194.0587	2.56	193/149/123	a
C8	4.14	C_21_H_20_O_12_	[M−H]−	Quercetin-3-o-glucopyranoside	463.0889	464.098	2.38	463/300/271/243/121	a, b, c
C9	4.47	C_11_H_10_O_4_	[M+H]+	Scoparone	207.0659	206.0593	0.74	207/192/179/151/135	a
C10	4.96	C_15_H_12_O_5_	[M+H]+	Naringenin	273.0772	272.0694	1.18	273/207/192/177/153	c
C11	5.02	C_17_H_24_O_9_	[M+Na]+	Syringoside	395.1312	372.1427	−1.9	369/298/232/192	a, b
C12	5.23	C_23_H_34_O_15_	[M+FA−H]−	Genipin 1-gentiobioside	595.1843	550.1938	4.95	549/224	a
C13	5.71	C_16_H_12_O_4_	[M−H]−	Isoformononetin	267.067	268.0747	1.08	267/252/225/207/147/123	b
C14	5.89	C_15_H_12_O_4_	[M+H]+	Isoliquiritigenin	257.0823	256.25338	0.87	257/147/119	c
C15	6.61	C_15_H_12_O_4_	[M−H]−	Liquiritigenin	255.0673	256.0743	0.67	255/165/135/119	c
C16	6.68	C_22_H_22_O_12_	[M−H]−	Cacticin	477.2361	478.1082	3.32	477/345/301/173	a
C17	7.66	C_25_H_24_O_12_	[M−H]−	3,5-O-Dicaffeoylquinic acid	515.1177	516.1275	4.43	515/493/475/353/255/179/123	a
C18	7.69	C_20_H_17_NO_5_	[M+H]+	Oxyberberine	352.1184	351.1088	1.71	352/336/322/320/308	b
C19	9.69	C_16_H_12_O_5_	[M+H]+	Genkwanin	285.0762	284.0693	0.92	285/269/257/207/147	c
C20	10.65	C_21_H_22_NO_4_	[M]+	Palmatine	352.1564	352.1598	0.01	352/336/322/308/294/278	b
C21	10.9	C_20_H_18_NO_4_	[M]+	Berberine	336.3283	336.1239	0.75	336/320/304/292/278	b
C22	11.58	C_30_H_46_O_4_	[M−H]−	18β-Glycyrrhetinic Acid	471.3478	470.68384	0.86	471/453/439/299/285/235/189/149/119	c
C23	12.16	C_21_H_22_O_5_	[M+H]+	Gancaonin I	355.1542	354.39638	1.09	355/335/283/121	c
C24	12.61	C_20_H_18_O_6_	[M−H]−	Licoisoflavone A	353.1044	354.1118	0.25	353/227/201/125/107	c
C25	12.63	C_20_H_16_O_5_	[M−H]−	Glabrone	335.0943	336.1011	4.17	337/319/295/269/254/210	c
C26	12.67	C_22_H_22_O_6_	[M−H]−	Licoricone	381.1353	382.1398	1.44	381/365/323/311/135	c
C27	12.76	C_20_H_16_O_6_	[M−H]−	Licoisoflavone B	351.0879	352.0967	1.28	353/311/299/283/153	c
C28	13.21	C_25_H_26_O_6_	[M−H]−	Glyasperin A	421.1663	422.1695	−1.82	421/366/281	c
C29	13.51	C_42_H_62_O_16_	[M−H]−	Glycyrrhizic acid	821.3971	822.405	1.97	821/759/645/551/449/351/289/193/113	c
C30	13.95	C_26_H_30_O_8_	[M+FA−H]−	Obaculactone	515.1924	470.1951	−3.46	381/229/137	b
C31	14.21	C_30_H_48_O_4_	[M−H]−	Hederagenin	471.347	472.355	1.19	471/419/303	c
C32	14.47	C_26_H_30_O_7_	[M−H]−	Obacunone	454.2002	454.1985	−0.25	337/285/243/201/161	b
C33	2.67	C_17_H_20_O_11_	[M+FA−H]−	Geniposide-C_6_H_10_O_5_(cleavage)-H2+C_6_H_8_O_6_	399.0935	400.1008	2.67	399/355/307/223	m
C34	3.23	C_17_H_22_O_11_	[M+FA−H]−	Geniposide+O-H_2_	401.1095	402.1168	1.6	369/305/270/123	m
C35	3.23	C_11_H_13_O_8_S	[M+FA−H]−	Geniposide-C_6_H_10_O_5_(cleavage)+SO_3_	305.0348	306.042	3.7	305/273/225/207/147/123	m
C36	3.43	C_17_H_24_O_11_	[M+FA−H]−	Geniposide+O	403.1246	404.1319	0.6	316/218/174/132	m
C37	3.74	C_26_H_34_O_12_	[M+FA−H]−	Obaculatone+2x(+H_2_O_2_)	537.1985	538.2058	1.3	507/447/361/331/181	m
C38	3.86	C_13_H_15_O_7_	[M+FA−H]−	Geniposide-C_6_H_10_O_5_(cleavage)+O+C_2_H_20_	283.0836	284.0909	4.5	283/265/187/107	m
C39	3.98	C_15_H_13_O_3_	[M+H]+	Isoliquiritigenin-O(cheavage)-H_2_	239.072	238.0647	7.2	239/137	m
C40	3.99	C_21_H_20_O_10_	[M+H]+	Isoliquiritigenin+C_6_H_8_O_6_	433.1146	432.1073	3.8	257/239/147/119	m
C41	4.08	C_21_H_19_O_10_	[M+H]+	Naringenin-O(cleavage)+C_6_H_8_O_6_	431.0987	432.1059	0.6	431/255/135	m
C42	4.97	C_21_H_20_O_12_	[M+H]+	Naringenin+C_6_H_8_O_6_	447.0942	448.1015	2	271/177/137/107	m
C43	6.16	C_17_H_21_O_9_	[M+FA−H]−	Geniposide-O(cleavage)-H_2_	369.12	370.1273	2.6	369/319/193/163/135	m
C44	6.6	C_15_H_10_O_4_	[M+H]+	Naringenin-O(cleavage)	255.0677	256.075	5.5	255/135/119	m
C45	7.23	C_16_H_22_O_8_	[M−H]−	Picrocrocinic acid+2x(-H_2_)	341.125	342.1323	2.7	165/150/121	m
C46	8.08	C_16_H_24_O_8_	[M−H]−	Picrocrocinic acid-H_2_	343.1411	344.1484	3.8	257/221/167/113	m
C47	8.93	C_20_H_15_NO_9_	[M+H]+	Oxyberberine+O-H_2_	366.0976	365.0904	1.3	337/308/291/227	m
C48	8.94	C_19_H_15_NO_7_	[M+H]+	Oxyberberine-CH2(cleavage)+2x (+O)	370.0931	369.0858	2.7	352/336/308/156	m
C49	9.68	C_20_H_15_NO_10_	[M+H]+	Oxyberberine+O	368.1139	367.1067	2.9	336/320/304/292	m
C50	11.29	C_20_H_17_NO_10_	[M+H]+	Oxyberberine+H_2_O_2_	386.1254	385.1181	5	195/175/123	m
C51	11.54	C_26_H_32_O_10_	[M+FA−H]−	Obaculatone+H_2_O_2_	503.1933	504.2005	1.9	333/239/191/113	m
C52	11.73	C_30_H_46_O_6_	[M+H]+	18β-Glycyrrhetinic acid+H_2_O_2_-H_2_	501.3199	502.3272	−4.4	471/385/309/253/187	m
C53	11.76	C_20_H_15_NO_11_	[M+H]+	Oxyberberine+H_2_O_2_-H_2_	384.1091	383.1018	3.6	384/184	m
C54	12.38	C_36_H_54_O_10_	[M+H]+	18β-Glycyrrhetinic acid+C6H8O6	645.3646	646.3719	0.3	570/469/355/287	m
C55	12.52	C_36_H_52_O_10_	[M+H]+	18β-Glycyrrhetinic acid-H_2_+C_6_H_8_O_6_	643.3493	644.3566	0.9	553/455/389/	m
C56	12.66	C_30_H_46_O_5_	[M+H]+	18β-Glycyrrhetinicacid+O	485.3289	486.3362	3.4	455/373/301/277	m
C57	12.83	C_30_H_44_O_5_	[M+H]+	18β-Glycyrrhetinic acid+O-H_2_	483.3129	484.3202	2.9	455/389/327/191	m
C58	14.12	C_30_H_44_O_4_	[M+H]+	18β-Glycyrrhetinic acid-H_2_	467.3175	468.3202	1.6	423/369/258/207	m
C59	15.86	C_30_H_45_O_3_	[M−H]−	Glycyrrhizic acid-C_12_H_16_O_13_(cleavage)	453.3375	454.3448	0.2	453/403	m

a, *Gardenia jasminoides* Ellis; b, *Phellodendronamurense*Rupr; c, *Glycyrrhiza uralensis* Fisch; m, metabolite.

### Correlation Analysis Between Biomarkers and Serum Constituents

**FIGURE 7:**
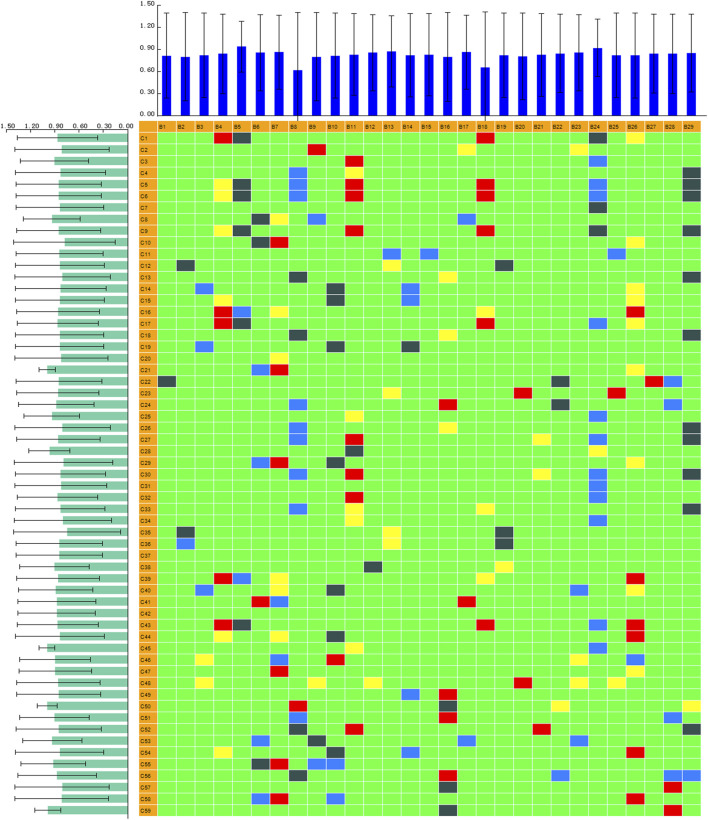
PCMS analysis of serum biomakers and chemical constituents in the ZBD group.

Chinmedomics provides a method to discover active constituents of ZBD that are absorbed *in vivo*. In establishing a correlation model between the metabolic biomarkers and chemical compounds, the relevant parameters were as follows: 0.9 < |r| ≤ 1 indicates an extremely high correlation, and 0.85 < |r| ≤ 0.9 indicates a high correlation. Using these parameters, the potentially active metabolites contributing to the main therapeutic effects of ZBD in the treatment of DHJS were discovered. The heatmap of the correlations between metabolic biomarkers and chemical compounds of ZBD absorbed *in vivo* is shown in Figure 7. The results showed that 10 compounds were significantly correlated with DHJS metabolite biomarkers, and these compounds were thus identified as the potential material basis of the therapeutic effects of ZBD against DHJS. The chemical compounds C13 (isoformononetin), C4 (3-O-feruloylquinic acid), C29 (glycyrrhizic acid), C33 (M1: geniposide- C_6_H_10_O_5_(cleavage)-H_2_+C_6_H_8_O_6_), C56 (M12: 18β-glycyrrhetinic acid+O), C15 (liquiritigenin), C50 (M23: oxyberberine+H_2_O_2_), C18 (oxyberberine), C53 (M26: Oxyberberine+H_2_O_2_-H_2_), and C30 (obaculactone) had extremely high correlations with the therapeutic effect of ZBD against DHJS. Therefore, the study results provide evidence for the potential effective constituents of ZBD against DHJS: these constituents were mostly correlated with the metabolite biomarkers B5 (estrone glucuronide), B18 (bilirubin), B4 (D-urobilin), B24 (LysoPC(18:1(9Z))), B11 (etiocholanedione), B26 (hematoporphyrin IX), B14 (7-ketodeoxycholic acid), B29 (biliverdin), B7 (sulfolithocholylglycine), B10 (3-oxo-4,6-choladienoic acid), and B8 (1b-hydroxycholic acid).

## Discussion

Preliminary experiments focused on behavioral, biochemical and liver histological indicators suggested the successful construction of the DHJS model in this study (Wang et al., 2012; Sun et al., 2019). A metabolomics method combined with UPLC-MS/MS was used to further study the changes in endogenous metabolites in DHJS rat serum. Our research group has mastered generating a DHJS mouse model (Heng et al., 2020) and has determined the procedure for modeling DHJS in rats through preliminary experiments. In the UPLC-MS/MS analysis, a QC sample was taken at every five samples, and five compounds with high response values in different retention periods were selected for QC analysis. The retention time and peak area RSDs of the selected compounds were less than 1.0%, indicating the reliability of this evaluation method. Using the pattern recognition data processing method, 42 biomarkers were preliminarily identified, and the metabolites that made the greatest contributions to differentiating the control and model groups were involved in 15 metabolic pathways. These experimental results showed that ZBD could significantly identify 29 biomarkers. ZBD plays an important role in the treatment of DHJS by affecting metabolic pathways such as primary bile acid synthesis, porphyrin and chlorophyll metabolism, and glycerophospholipid metabolism.

Primary bile acids are biosynthesized, and the liver plays an important role in bile acid metabolism, which is closely related to bile acid synthesis, secretion and transformation. Changes in bile acids are considered metabolomic markers of liver injury (Shen and Wei, 2019). Primary bile acids, cholic acids and chenodeoxyglycocholic acid are synthesized in the liver, combined with taurine or glycine, and then secreted into the intestinal tract through bile (Russell, 2003; Hofmann and Hagey, 2008). This study showed that the contents of taurocholic acid, cholic acid and glycocholic acid, indicators of primary bile acid biosynthesis, were higher in DHJS rats than in control rats; this result may be related to competition with bilirubin for binding to albumin and inhibition of normal bile acid metabolism. The increase in free bilirubin content led to the occurrence of DHJS.

Porphyrin and chlorophyll metabolism generates bilirubin as the main metabolite of iron porphyrin compounds in the body. There are two types of bilirubin in the blood: the single or double glucuronic acid (ester) and the free type, which is the normal metabolite of hemoglobin (Bonkovsky et al., 2013). Heme, an iron porphyrin compound, is an auxiliary hemoglobin group that is converted to biliverdin, carbon monoxide and iron by heme oxygenase and then reduced to bilirubin (Gibbons et al., 2019). The results of this study showed that D-urobilin levels were higher in the model group than in the control group, indicating the presence of disordered porphyrin and chlorophyll metabolism. The ZBD group showed significant regulation of the levels of the metabolites bilirubin, biliverdin and D-urobilin. DHJS can be treated by affecting hemoglobin synthesis, heme decomposition, biliverdin reduction and bilirubin metabolism.

Lipid metabolism is one of the important physiological functions of the liver (Mashek, 2021). Bile acids, lysolecithin, and unsaturated fatty acids are the core metabolites of the liver (Narwal et al., 2019). Bile acids contribute to lipid emulsification, enhance pancreatic lipolysis, and promote intestinal absorption of lipids by forming mixed gels to improve lipid solubility (Taoka et al., 2016). Liver tissue lesions occurred and the level of taurocholic acid was increased in the model group, causing abnormalities in bile acid metabolism and subsequently inducing abnormalities in lipid metabolism, so the levels of lipid molecules (PC(18:4(6Z,9Z,12Z, 15Z)/P-18:1(11Z)), LysoPC(18:1(9Z)), PC(16:0/16:0) in the blood were reduced, exacerbating bile stasis, and after administration of ZBD treatment, the content level of lipidable molecules increased, and DHJS was treated by regulating glycerophospholipid metabolism.

According to the theory and method of serum pharmacochemistry (Zhang et al., 2013a), drugs enter the blood, get metabolized and distributed and then produce specific biological effects through specific mechanisms. Blood components are the ultimate “effective components.” We characterized 59 final effective components of ZBD *in vivo* and preliminarily identified 10 substances that were highly correlated with drug efficacy. These 10 compounds were extracted from three components of ZBD. According to the chinmedomics analysis, the highly correlated blood components included the metabolite geniposide from *Gardenia jasminoides* Ellis., berberine oxide and its metabolites from *Phellodendron amurense* Rupr., and isoliquiritigenin from *Glycyrrhiza uralensis* Fisch.

Geniposide have hepatoprotective and hypolipidemic effects in cholestasis model rats, which may be achieved through the regulation of bile acid transporter MRP2 and BSEP function by the nuclear receptor FXR (Liu et al., 2020). And attenuated ANIT-induced hepatotoxicity and cholestasis in rats by inhibiting OATP2 protein, while activating FXR, PXR and SHP receptor protein expression and reducing the regulation of basolateral bile acid uptake, thereby ameliorating cholestasis in model rats (Zhang et al., 2013b; Wang et al., 2017). Glycyrrhetinic acid is a pentacyclic triterpenoid, which is one of the main active ingredients of *Glycyrrhiza uralensis* Fisch., and its derivatives have various pharmacological activities such as anti-inflammatory, anti-viral and anti-tumor (Wu et al., 2018). Oxyberberine has significant therapeutic effects in rats with nonalcoholic liver injury. Oxyberberine has a strong affinity for AMP-dependent protein kinase (AMPK) *in vivo* and hyperphosphorylates AMPK better than berberine *in vivo*, resulting in increased ACC synthase mRNA expression in the liver and increased UCP-1 protein expression in adipose tissue (Li et al., 2021).It is speculated that these compounds are potentially active metabolites for ZBD in treating DHJS *in vivo*.

Chinmedomics combined with UPLC-MS/MS was used to study the targets and effective constituents of ZBD in the context of DHJS. Chinmedomics provides a method to clarify the efficacy of TCM: it offers a connection between the absorbed chemical components and metabolic biomarkers and enables the calculation of the correlation coefficient of each component by using an established mathematical model to evaluate the effective components of TCM.

## Data Availability

The original contributions presented in the study are included in the article/Supplementary Materials, further inquiries can be directed to the corresponding author.
