# Supplementary material for: Targets and Effective Constituents of ZhiziBaipi Decoction for Treating Damp-Heat Jaundice Syndrome Based on Chinmedomics Coupled with UPLC-MS/MS
Source: Front Pharmacol. 2022 Apr 5;13:857361. doi: 10.3389/fphar.2022.857361 (PMC9016223; doi:10.3389/fphar.2022.857361)
Supplement: Supplementary file 1 [file Table1.docx]

Supplementary Material

**Table 1. Detailed information on biomarkers tentatively identified by serum metabolomics**

| NO. | RT  min | M/Z  Determined | Scan Mode | Proposed composition | Postulated Identity | Model vs Control | ZBD vs Model |
| --- | --- | --- | --- | --- | --- | --- | --- |
| 1(B1) | 0.59 | 762.537 | ESI- | C_44_H_78_NO_7_P | PC(18:4(6Z,9Z,12Z,15Z)/P-18:1(11Z)) | ↓ | ↑ |
| 2 | 1.69 | 219.0774 | ESI- | C_11_H_12_N_2_O_3_ | 5-Hydroxy-L-tryptophan | ↓ |  |
| 3(B2) | 1.71 | 258.0089 | ESI- | C_8_H_7_NO_4_S | Indoxyl sulfate | ↓ | ↑ |
| 4(B3) | 2.01 | 613.2982 | ESI+ | C_33_H_42_N_4_O_6_ | D-Urobilinogen | ↑ | ↓ |
| 5(B4) | 2.06 | 611.2817 | ESI- | C_33_H_40_N_4_O_6_ | D-Urobilin | ↑ | ↓ |
| 6(B5) | 2.19 | 445.1898 | ESI- | C_24_H_30_O_8_ | Estrone glucuronide | ↓ | ↑ |
| 7(B6) | 2.28 | 199.022 | ESI+ | C_6_H_8_O_6_ | D-Glucurono-6,3-lactone | ↑ | ↓ |
| 8(B7) | 2.61 | 512.2682 | ESI- | C_26_H_43_NO_7_S | Sulfolithocholylglycine | ↑ | ↓ |
| 9(B8) | 2.67 | 423.2726 | ESI- | C_24_H_40_O_6_ | 1b-Hydroxycholic acid | ↓ | ↑ |
| 10(B9) | 2.83 | 514.2841 | ESI- | C_26_H_45_NO_7_S | Taurocholic acid | ↑ | ↓ |
| 11 | 2.98 | 448.3064 | ESI- | C_26_H_43_NO_5_ | Chenodeoxyglycocholic acid | ↑ |  |
| 12 | 2.98 | 464.3015 | ESI- | C_26_H_43_NO_6_ | Glycocholic acid | ↑ |  |
| 13(B10) | 3.05 | 371.2558 | ESI+ | C_24_H_34_O_3_ | 3-Oxo-4,6-choladienoic acid | ↑ | ↓ |
| 14(B11) | 3.05 | 333.204 | ESI- | C_19_H_28_O_2_ | Etiocholanedione | ↓ | ↑ |
| 15(B12) | 3.06 | 148.043 | ESI- | C_5_H_11_NO_2_S | L-Methionine | ↑ | ↓ |
| 16(B13) | 3.08 | 471.2418 | ESI- | C_24_H_40_O_7_S | Chenodeoxycholic acid sulfate | ↑ | ↓ |
| 17(B14) | 3.1 | 405.264 | ESI- | C_24_H_38_O_5_ | 7-Ketodeoxycholic acid | ↓ | ↑ |
| 18(B15) | 3.25 | 498.2892 | ESI- | C_26_H_45_NO_6_S | Tauroursodeoxycholic acid | ↑ | ↓ |
| 19 | 3.25 | 482.2913 | ESI- | C_26_H_45_NO_5_S | Lithocholyltaurine | ↑ |  |
| 20 | 3.28 | 431.2765 | ESI+ | C_24_H_40_O_5_ | Cholic acid | ↓ |  |
| 21(B16) | 3.5 | 389.2687 | ESI- | C_24_H_38_O_4_ | 12-Ketodeoxycholic acid | ↑ | ↓ |
| 22(B17) | 3.56 | 551.3196 | ESI- | C_30_H_48_O_9_ | Lithocholate 3-O-glucuronide | ↓ | ↑ |
| 23(B18) | 3.59 | 583.2558 | ESI- | C_33_H_36_N_4_O_6_ | Bilirubin | ↑ | ↓ |
| 24 | 3.85 | 378.2413 | ESI- | C_18_H_38_NO_5_P | Sphingosine 1-phosphate | ↑ |  |
| 25(B19) | 3.91 | 311.2196 | ESI- | C_18_H_32_O_4_ | 13-L-Hydroperoxylinoleic acid | ↑ | ↓ |
| 26(B20) | 3.99 | 391.2847 | ESI- | C_24_H_40_O_4_ | 3b,12a-Dihydroxy-5a-cholanoic acid | ↑ | ↓ |
| 27(B21) | 4.02 | 562.3144 | ESI- | C_26_H_48_NO_7_P | LysoPC(18:3(9Z,12Z,15Z)) | ↑ | ↓ |
| 28 | 4.13 | 285.2065 | ESI- | C_16_H_30_O_4_ | Hexadecanedioic acid | ↓ |  |
| 29(B22) | 4.29 | 429.2993 | ESI- | C_27_H_42_O_4_ | 7alpha-Hydroxy-3-oxo-4-cholestenoate | ↓ | ↑ |
| 30 | 4.69 | 459.2491 | ESI+ | C_21_H_41_O_7_P | DHAP(18:0) | ↓ |  |
| 31 | 4.87 | 191.0181 | ESI+ | C_5_H_4_N_4_O_3_ | Uric acid | ↑ |  |
| 32(B23) | 4.88 | 624.3413 | ESI- | C_32_H_51_NO_11_ | Glycochenodeoxycholic acid 3-glucuronide | ↓ | ↑ |
| 33(B24) | 4.89 | 522.3561 | ESI+ | C_26_H_52_NO_7_P | LysoPC(18:1(9Z)) | ↓ | ↑ |
| 34(B25) | 4.98 | 471.3468 | ESI+ | C_28_H_48_O_4_ | 2-Deoxycastasterone | ↓ | ↑ |
| 35(B26) | 5.17 | 599.2885 | ESI+ | C_34_H_38_N_4_O_6_ | Hematoporphyrin IX | ↓ | ↑ |
| 36(B27) | 5.62 | 555.2942 | ESI+ | C_27_H_48_O_8_S | 5b-Cyprinol sulfate | ↓ | ↑ |
| 37(B28) | 5.72 | 445.3309 | ESI- | C_27_H_44_O_2_ | 7a-Hydroxy-cholestene-3-one | ↑ | ↓ |
| 38 | 6.2 | 345.2042 | ESI- | C_21_H_30_O_4_ | Corticosterone | ↑ |  |
| 39 | 6.63 | 756.5532 | ESI+ | C_40_H_80_NO_8_P | PC(16:0/16:0) | ↓ |  |
| 40 | 6.64 | 184.0741 | ESI+ | C_10_H_11_NO | Tryptophanol | ↓ |  |
| 41 | 6.98 | 367.158 | ESI- | C_19_H_28_O_5_S | Dehydroepiandrosterone sulfate | ↑ |  |
| 42(B29) | 7.79 | 581.2401 | ESI- | C_33_H_34_N_4_O_6_ | Biliverdin | ↓ | ↑ |

↑ and ↓ represent higher and lower level; Model vs control: Model group compared with control group; ZBD vs model: ZBD group compared with model group. No. B1-B29 correspond to Figure 7.

Table 2. Analysis of constituents in DHJS rat serum after the oral administration of ZBD

| **NO.** | **RT/min** | **Formula** | **Ion mode** | **Compound name** | **Measured mass/Da** | **Theoretical mass /Da** | **Error/Da** | **Fragments** | **Origin** |
| --- | --- | --- | --- | --- | --- | --- | --- | --- | --- |
| C1 | 2.88 | C_27_H_32_O_14_ | [M-H]- | Naringin | 579.172 | 580.1799 | 0.82 | 580/549/417/353/255/191 | c |
| C2 | 3.08 | C_27_H_30_O_15_ | [M-H]- | Nicotiflorin | 593.1522 | 594.1618 | 1.35 | 593/549/475/137 | c |
| C3 | 3.31 | C_20_H_21_NO_4_ | [M+H]+ | Tetrahydroberberine | 340.1556 | 339.1494 | 4.06 | 340/309/276/147 | b |
| C4 | 3.49 | C_17_H_20_O_9_ | [M-H]- | 3-O-Feruloylquinic acid | 367.1044 | 368.1115 | -2.93 | 367/191/173/111 | b |
| C5 | 4.01 | C_21_H_22_O_9_ | [M-H]- | Isoliquiritin | 417.121 | 418.1308 | 0.77 | 417/255/135/119 | c |
| C6 | 4.11 | C_11_H_12_O_5_ | [M-H]- | Sinapic acid | 223.0622 | 224.0702 | 0.69 | 225/207/189/167/147 | c |
| C7 | 4.12 | C_10_H_10_O_4_ | [M-H]- | Ferulic acid | 193.0511 | 194.0587 | 2.56 | 193/149/123 | a |
| C8 | 4.14 | C_21_H_20_O_12_ | [M-H]- | Quercetin-3-o-glucopyranoside | 463.0889 | 464.098 | 2.38 | 463/300/271/243/121 | a,b,c |
| C9 | 4.47 | C_11_H_10_O_4_ | [M+H]+ | Scoparone | 207.0659 | 206.0593 | 0.74 | 207/192/179/151/135 | a |
| C10 | 4.96 | C_15_H_12_O_5_ | [M+H]+ | Naringenin | 273.0772 | 272.0694 | 1.18 | 273/207/192/177/153 | c |
| C11 | 5.02 | C_17_H_24_O_9_ | [M+Na]+ | Syringoside | 395.1312 | 372.1427 | -1.9 | 369/298/232/192 | a,b |
| C12 | 5.23 | C_23_H_34_O_15_ | [M+FA-H]- | Genipin 1-gentiobioside | 595.1843 | 550.1938 | 4.95 | 549/224 | a |
| C13 | 5.71 | C_16_H_12_O_4_ | [M-H]- | Isoformononetin | 267.067 | 268.0747 | 1.08 | 267/252/225/207/147/123 | b |
| C14 | 5.89 | C_15_H_12_O_4_ | [M+H]+ | Isoliquiritigenin | 257.0823 | 256.25338 | 0.87 | 257/147/119 | c |
| C15 | 6.61 | C_15_H_12_O_4_ | [M-H]- | Liquiritigenin | 255.0673 | 256.0743 | 0.67 | 255/165/135/119 | c |
| C16 | 6.68 | C_22_H_22_O_12_ | [M-H]- | Cacticin | 477.2361 | 478.1082 | 3.32 | 477/345/301/173 | a |
| C17 | 7.66 | C_25_H_24_O_12_ | [M-H]- | 3,5-O-Dicaffeoylquinic acid | 515.1177 | 516.1275 | 4.43 | 515/493/475/353/255/179/123 | a |
| C18 | 7.69 | C_20_H_17_NO_5_ | [M+H]+ | Oxyberberine | 352.1184 | 351.1088 | 1.71 | 352/336/322/320/308 | b |
| C19 | 9.69 | C_16_H_12_O_5_ | [M+H]+ | Genkwanin | 285.0762 | 284.0693 | 0.92 | 285/269/257/207/147 | c |
| C20 | 10.65 | C_21_H_22_NO_4_ | [M]+ | Palmatine | 352.1564 | 352.1598 | 0.01 | 352/336/322/308/294/278 | b |
| C21 | 10.9 | C_20_H_18_NO_4_ | [M]+ | Berberine | 336.3283 | 336.1239 | 0.75 | 336/320/304/292/278 | b |
| C22 | 11.58 | C_30_H_46_O_4_ | [M-H]- | 18β-Glycyrrhetinic Acid | 471.3478 | 470.68384 | 0.86 | 471/453/439/299/285/235/189/149/119 | c |
| C23 | 12.16 | C_21_H_22_O_5_ | [M+H]+ | Gancaonin I | 355.1542 | 354.39638 | 1.09 | 355/335/283/121 | c |
| C24 | 12.61 | C_20_H_18_O_6_ | [M-H]- | Licoisoflavone A | 353.1044 | 354.1118 | 0.25 | 353/227/201/125/107 | c |
| C25 | 12.63 | C_20_H_16_O_5_ | [M-H]- | Glabrone | 335.0943 | 336.1011 | 4.17 | 337/319/295/269/254/210 | c |
| C26 | 12.67 | C_22_H_22_O_6_ | [M-H]- | Licoricone | 381.1353 | 382.1398 | 1.44 | 381/365/323/311/135 | c |
| C27 | 12.76 | C_20_H_16_O_6_ | [M-H]- | Licoisoflavone B | 351.0879 | 352.0967 | 1.28 | 353/311/299/283/153 | c |
| C28 | 13.21 | C_25_H_26_O_6_ | [M-H]- | Glyasperin A | 421.1663 | 422.1695 | -1.82 | 421/366/281 | c |
| C29 | 13.51 | C_42_H_62_O_16_ | [M-H]- | Glycyrrhizic acid | 821.3971 | 822.405 | 1.97 | 821/759/645/551/449/351/289/193/113 | c |
| C30 | 13.95 | C_26_H_30_O_8_ | [M+FA-H]- | Obaculactone | 515.1924 | 470.1951 | -3.46 | 381/229/137 | b |
| C31 | 14.21 | C_30_H_48_O_4_ | [M-H]- | Hederagenin | 471.347 | 472.355 | 1.19 | 471/419/303 | c |
| C32 | 14.47 | C_26_H_30_O_7_ | [M-H]- | Obacunone | 454.2002 | 454.1985 | -0.25 | 337/285/243/201/161 | b |
| C33 | 2.67 | C_17_H_20_O_11_ | [M+FA-H]- | Geniposide-C_6_H_10_O_5_(cleavage)-H2+C_6_H_8_O_6_ | 399.0935 | 400.1008 | 2.67 | 399/355/307/223 | m |
| C34 | 3.23 | C_17_H_22_O_11_ | [M+FA-H]- | Geniposide+O-H_2_ | 401.1095 | 402.1168 | 1.6 | 369/305/270/123 | m |
| C35 | 3.23 | C_11_H_13_O_8_S | [M+FA-H]- | Geniposide-C_6_H_10_O_5_(cleavage)+SO_3_ | 305.0348 | 306.042 | 3.7 | 305/273/225/207/147/123 | m |
| C36 | 3.43 | C_17_H_24_O_11_ | [M+FA-H]- | Geniposide+O | 403.1246 | 404.1319 | 0.6 | 316/218/174/132 | m |
| C37 | 3.74 | C_26_H_34_O_12_ | [M+FA-H]- | Obaculatone+2x(+H_2_O_2_) | 537.1985 | 538.2058 | 1.3 | 507/447/361/331/181 | m |
| C38 | 3.86 | C_13_H_15_O_7_ | [M+FA-H]- | Geniposide-C_6_H_10_O_5_(cleavage)+O+C_2_H_20_ | 283.0836 | 284.0909 | 4.5 | 283/265/187/107 | m |
| C39 | 3.98 | C_15_H_13_O_3_ | [M+H]+ | Isoliquiritigenin-O(cheavage)-H_2_ | 239.072 | 238.0647 | 7.2 | 239/137 | m |
| C40 | 3.99 | C_21_H_20_O_10_ | [M+H]+ | Isoliquiritigenin+C_6_H_8_O_6_ | 433.1146 | 432.1073 | 3.8 | 257/239/147/119 | m |
| C41 | 4.08 | C_21_H_19_O_10_ | [M+H]+ | Naringenin-O(cleavage)+C_6_H_8_O_6_ | 431.0987 | 432.1059 | 0.6 | 431/255/135 | m |
| C42 | 4.97 | C_21_H_20_O_12_ | [M+H]+ | Naringenin+C_6_H_8_O_6_ | 447.0942 | 448.1015 | 2 | 271/177/137/107 | m |
| C43 | 6.16 | C_17_H_21_O_9_ | [M+FA-H]- | Geniposide-O(cleavage)-H_2_ | 369.12 | 370.1273 | 2.6 | 369/319/193/163/135 | m |
| C44 | 6.6 | C_15_H_10_O_4_ | [M+H]+ | Naringenin-O(cleavage) | 255.0677 | 256.075 | 5.5 | 255/135/119 | m |
| C45 | 7.23 | C_16_H_22_O_8_ | [M-H]- | Picrocrocinic acid+2x(-H_2_) | 341.125 | 342.1323 | 2.7 | 165/150/121 | m |
| C46 | 8.08 | C_16_H_24_O_8_ | [M-H]- | Picrocrocinic acid-H_2_ | 343.1411 | 344.1484 | 3.8 | 257/221/167/113 | m |
| C47 | 8.93 | C_20_H_15_NO_9_ | [M+H]+ | Oxyberberine+O-H_2_ | 366.0976 | 365.0904 | 1.3 | 337/308/291/227 | m |
| C48 | 8.94 | C_19_H_15_NO_7_ | [M+H]+ | Oxyberberine-CH2(cleavage)+2x(+O) | 370.0931 | 369.0858 | 2.7 | 352/336/308/156 | m |
| C49 | 9.68 | C_20_H_15_NO_10_ | [M+H]+ | Oxyberberine+O | 368.1139 | 367.1067 | 2.9 | 336/320/304/292 | m |
| C50 | 11.29 | C_20_H_17_NO_10_ | [M+H]+ | Oxyberberine+H_2_O_2_ | 386.1254 | 385.1181 | 5 | 195/175/123 | m |
| C51 | 11.54 | C_26_H_32_O_10_ | [M+FA-H]- | Obaculatone+H_2_O_2_ | 503.1933 | 504.2005 | 1.9 | 333/239/191/113 | m |
| C52 | 11.73 | C_30_H_46_O_6_ | [M+H]+ | 18β-Glycyrrhetinic acid+H_2_O_2_-H_2_ | 501.3199 | 502.3272 | -4.4 | 471/385/309/253/187 | m |
| C53 | 11.76 | C_20_H_15_NO_11_ | [M+H]+ | Oxyberberine+H_2_O_2_-H_2_ | 384.1091 | 383.1018 | 3.6 | 384/184 | m |
| C54 | 12.38 | C_36_H_54_O_10_ | [M+H]+ | 18β-Glycyrrhetinic acid+C6H8O6 | 645.3646 | 646.3719 | 0.3 | 570/469/355/287 | m |
| C55 | 12.52 | C_36_H_52_O_10_ | [M+H]+ | 18β-Glycyrrhetinic acid-H_2_+C_6_H_8_O_6_ | 643.3493 | 644.3566 | 0.9 | 553/455/389/ | m |
| C56 | 12.66 | C_30_H_46_O_5_ | [M+H]+ | 18β-Glycyrrhetinicacid+O | 485.3289 | 486.3362 | 3.4 | 455/373/301/277 | m |
| C57 | 12.83 | C_30_H_44_O_5_ | [M+H]+ | 18β-Glycyrrhetinic acid+O-H_2_ | 483.3129 | 484.3202 | 2.9 | 455/389/327/191 | m |
| C58 | 14.12 | C_30_H_44_O_4_ | [M+H]+ | 18β-Glycyrrhetinic acid-H_2_ | 467.3175 | 468.3202 | 1.6 | 423/369/258/207 | m |
| C59 | 15.86 | C_30_H_45_O_3_ | [M-H]- | Glycyrrhizic acid-C_12_H_16_O_13_(cleavage) | 453.3375 | 454.3448 | 0.2 | 453/403 | m |

Note: a: *Gardenia jasminoides* Ellis. b:*Phellodendronamurense*Rupr. c:*Glycyrrhiza uralensis* Fisch. m: metabolite.
